# Cervical Cancer Screening, HPV Vaccination, and Cervical Cancer Elimination

**DOI:** 10.1001/jamanetworkopen.2025.26683

**Published:** 2025-08-12

**Authors:** Xuan Quy Luu, Jae Kwan Jun, Mina Suh, Jin-Kyoung Oh, Su-Yeon Yu, Kui Son Choi

**Affiliations:** 1 Graduate School of Cancer Science and Policy, National Cancer Center, Goyang, South Korea; 2 Department of Biostatistics, Faculty of Fundamental Science, Hanoi University of Public Health, Hanoi, Vietnam; 3 National Cancer Control Institute, National Cancer Center, Goyang, South Korea; 4 College of Pharmacy, Kangwon National University, Chuncheon, South Korea; 5 Department of Medical Information College of Nursing and Health, Kongju National University, South Korea

## Abstract

**Importance:**

To accelerate cervical cancer elimination, it is essential to evaluate the combined impact of ongoing screening and human papillomavirus (HPV) vaccination efforts, as the disease remains a significant global public health concern.

**Objective:**

To estimate how different cervical cancer screening strategies, along with HPV vaccination, affect the cervical cancer burden in South Korea.

**Design, Setting, and Participants:**

This decision analytical model used a deterministic age-structured dynamic model to simulate HPV transmission and cervical cancer progression, considering sexual behavior and population structure, for the Korean population from 2024 to 2100. The study was conducted from April 2023 to September 2024.

**Exposures:**

Cervical cancer screening using Papanicolaou (Pap) or HPV testing, with HPV vaccination.

**Main Outcomes and Measures:**

The cumulative number of incident cases of cervical cancer and deaths for the projected period are reported.

**Results:**

The study modeled more than 51 million Korean residents, with 25.88 million women (50.1%) and 10.05 million women aged 18 to 49 years. Among Pap test–based strategies, biennial screening for women aged 20 years and older was associated with the lowest number of cervical cancer cases (47 358) and deaths (34 242) by 2100, with as much as a 10% mortality reduction compared with other Pap test–based strategies. High-risk HPV (hrHPV) testing every 2 or 3 years was estimated to further reduce cases by as much as 27% and deaths by 13%. Depending on the screening strategy, cervical cancer elimination based on the World Health Organization definition (4 cases per 100 000 women) could occur as early as 2038 (with 2-year hrHPV testing starting at age 25 years or older) or as late as 2055 (5-year Pap testing for individuals aged 25 to 64 years). The current screening policy (Pap test every 2 years starting at age 20 years) was estimated to reach disease elimination by 2044. In an ideal scenario with 70% screening and 90% vaccination coverage from 2030, an additional 12% of cases and 7% of deaths could be prevented. Strategies using a 2-year interval for hrHPV testing could lead to the earliest cervical cancer elimination by 2034.

**Conclusions and Relevance:**

In this decision analytical model, an ideal scenario with higher cervical cancer screening and HPV vaccination rates was associated with substantially reduced cervical cancer incidence and mortality and accelerated elimination in South Korea. Notably, hrHPV testing with 2- or 3-year screening intervals was estimated to achieve elimination as early as 2034.

## Key Points

**Question:**

What are the estimated effects of different cervical cancer screening strategies, along with human papillomavirus (HPV) vaccination, on the goal of cervical cancer elimination?

**Findings:**

This decision analytical model, which simulated the Korean population from 2024 to 2100, found that under the current screening policy (2-year Papanicolaou testing started at age 20 years), the incidence rate was expected to decline below the World Health Organization disease elimination threshold of 4 cases per 100 000 women by 2044. In an ideal scenario with 70% screening and 90% vaccination rates, high-risk HPV testing with a 2-year interval starting at age 20 years was estimated to lead to elimination by 2034.

**Meaning:**

These findings suggest that implementing high-risk HPV testing and increasing HPV vaccination rates could accelerate cervical cancer elimination.

## Teaser

This decision analytical model study examines how different strategies for Papanicolaou testing, human papillomavirus (HPV) testing, and HPV vaccination are associated with reducing the burden of cervical cancer to 4 cases per 100 000 women in South Korea.

## Introduction

Cervical cancer represents a global health issue. In 2020, approximately 604 127 new cervical cancer cases were reported globally, and the age-standardized incidence rate (ASR) was 13.3 cases per 100 000 women-years.^1^ While the incidence rate has decreased in high-income countries, disparities still persist, with more than 85% of new cases and deaths being concentrated in lower-resource regions.^1,2^

In 2018, the World Health Organization (WHO) announced a global call for action,^3^ culminating in the November 2020 launch of the global strategy to accelerate the elimination of cervical cancer.^4^ This landmark strategy aims for an incidence rate of fewer than 4 cases per 100 000 women, targeting 90% human papillomavirus (HPV) vaccination coverage among girls by age 15 years, 70% screening coverage for women by ages 35 and 45 years, and 90% treatment provision for diagnosed cases.^4^

While cervical cancer remains a public health concern in South Korea, the ASR of cervical cancer has halved over the last 20 years, reaching 8 cases per 100 000 women in 2019.^5^ This positive trend can largely be attributed to the implementation of the National Cancer Screening Program (NCSP) for cervical cancer in 1999, which provided biennial Papanicolaou (Pap) tests to women aged 20 years or older.^6^ Over time, the screening rate remained relatively stable, with the rates of 56.0% in 2005 and 57.1% in 2020.^6^ Additionally, the introduction of HPV vaccination in the National Immunization Program (NIP) in 2016, which targeted girls aged 12 years with 2-dose regimens of 2-valent or 4-valent HPV vaccines, resulted in an overall vaccination coverage of approximately 74% by 2023.^7^

While South Korea has significantly reduced its cervical cancer burden, analyses on when the nation can realistically achieve the WHO elimination threshold and what additional strategies could expedite this are limited. Therefore, using a dynamic modeling approach, we estimated the association of various cervical cancer screening and HPV vaccination strategies with future incidence and mortality and projected the potential elimination year for South Korea.

## Methods

### Model Overview and Parameterization

We used a deterministic age-structured dynamic model commonly used to simulate the transmission of HPV infection and its progression to cervical cancer.^8,9,10,11^ This model integrates multiple components, including HPV transmission incorporated with local sexual behavior patterns, cervical cancer progression, and effects of vaccination and screening interventions. For the HPV transmission component, we adopted a 3-stage susceptible-infected-removed (SIR) structure reflecting transitions between health states based on contact patterns, sexual behavior, and immunity status. Additionally, to simulate the effect of health interventions, the model was extended to account for the HPV vaccination stage and natural history of cervical cancer with cervical cancer screening. The natural progression of cervical cancer was modeled through the stages of cervical intraepithelial neoplasia (CIN) 1, CIN 2, and CIN 3, leading to invasive cervical cancer. Invasive cancer was classified based on the Surveillance, Epidemiology, and End Results summary stages of localized, regional, and distant cancers (eFigure 1 in Supplement 1).^12^ By simulating these components, we could capture the effects of both vaccination and screening in reducing the cervical cancer burden.

Our population was stratified by sex and age, with sexual mixing between subpopulations (age groups and sex) to reflect age- and sex-specific contact rates. We assumed independent transmission dynamics of different oncogenic HPV subtypes and ran separate models for each. The key model assumptions are presented in eTable 1 in Supplement 1, whereas the model equations are provided in the eAppendix in Supplement 1. The current study was approved by the institutional review board of the National Cancer Center, Korea, and informed consent was waived due to the use of summarized, secondary data. The study was conducted from April 2023 to September 2024. The information in the study was reported according to the Consolidated Health Economic Evaluation Reporting Standards 2022 (CHEERS) guideline and HPV-FRAME for the analytic model.^20,21^

### Cervical Cancer Screening and Vaccination Strategies

We simulated 36 different screening strategies in combination with 1 vaccination strategy (2-valent or 4-valent HPV vaccines for 12-year-old girls) following the NIP.^7^ Screening strategies varied according to the starting age (20 or 25 years), screening interval (2, 3, or 5 years), and test type, including both Pap and high-risk HPV (hrHPV) testing. These combinations were selected to reflect historically recommended and policy-relevant options in South Korea as well as in other countries and regions. Detailed characteristics of the screening strategies are summarized in eTable 2 in Supplement 1.

### Model Scenarios and Outcome Measures

The model was run under 2 scenarios to reflect real and ideal situations. The realistic scenario assumed the current cervical cancer screening rate (51.5%) and vaccination rate based on data from the NCSP and NIP.^13^ According to a report from the NIP, the 2-dose vaccination rates among girls born in 2009 and 2010 were 76.0% and 76.6% by 2022 and 2023, respectively.^13^ Therefore, in the current study’s base scenario, the complete vaccination rate for girls was set at 75%. The ideal scenario assumes an increase in the screening rate to 70% and vaccination rate to 90% from 2030 onward, reflecting the WHO’s target for cervical cancer elimination. For both scenarios, the model simulated the population of women in South Korea from 2024 to 2100 and its dynamics to obtain cervical incident cases and deaths for the projected period. The time of cervical cancer elimination was defined as an annual ASR of fewer than 4 cervical cancer cases per 100 000 women (2020 South Korean Standard Population).

### Model Calibration

To estimate parameters associated with cervical cancer, we used the goodness-of-fit method based on the sum-of-squares function to align our model with 2 main sources of epidemiological data: (1) data on the prevalence of HPV infection collected from a large Korean cohort study involving 18 170 women^14^ and (2) data on the cervical cancer incidence collected from 2010 to 2019 provided by the Korea Center Cancer Registry (KCCR).^15^ The parameter ranges were determined based on the epidemiological characteristics of the disease or condition and previous literature.^16,17,18,19^

### Sensitivity Analysis

We performed a 1-way sensitivity analysis to assess the key factors affecting the estimated year of cervical cancer elimination in Korea. The factors considered included the absence of an HPV vaccine in the NIP, opportunistic screening rates, inclusion of boys in the NIP for HPV vaccination, cross-protection of HPV vaccination against other hrHPV types, and population size stability. The effects of using the WHO World Standard Population for the ASR were also examined. The detailed assumptions for each sensitivity analysis are listed in eTable 3 in Supplement 1.

### Statistical Analysis

The model was built and analyzed using R version 4.2.1 (R Project for Statistical Computing). Additional analyses combining the cervical cancer burden from different HPV subtypes and standardizing age-specific incidence rates were conducted using SAS version 9.1 (SAS Institute Inc) and Excel version 2506 (Microsoft Corp). A comprehensive list of the model input parameters is provided in eTable 4 in Supplement 1.

## Results

### Model Calibration

**Figure 1.**
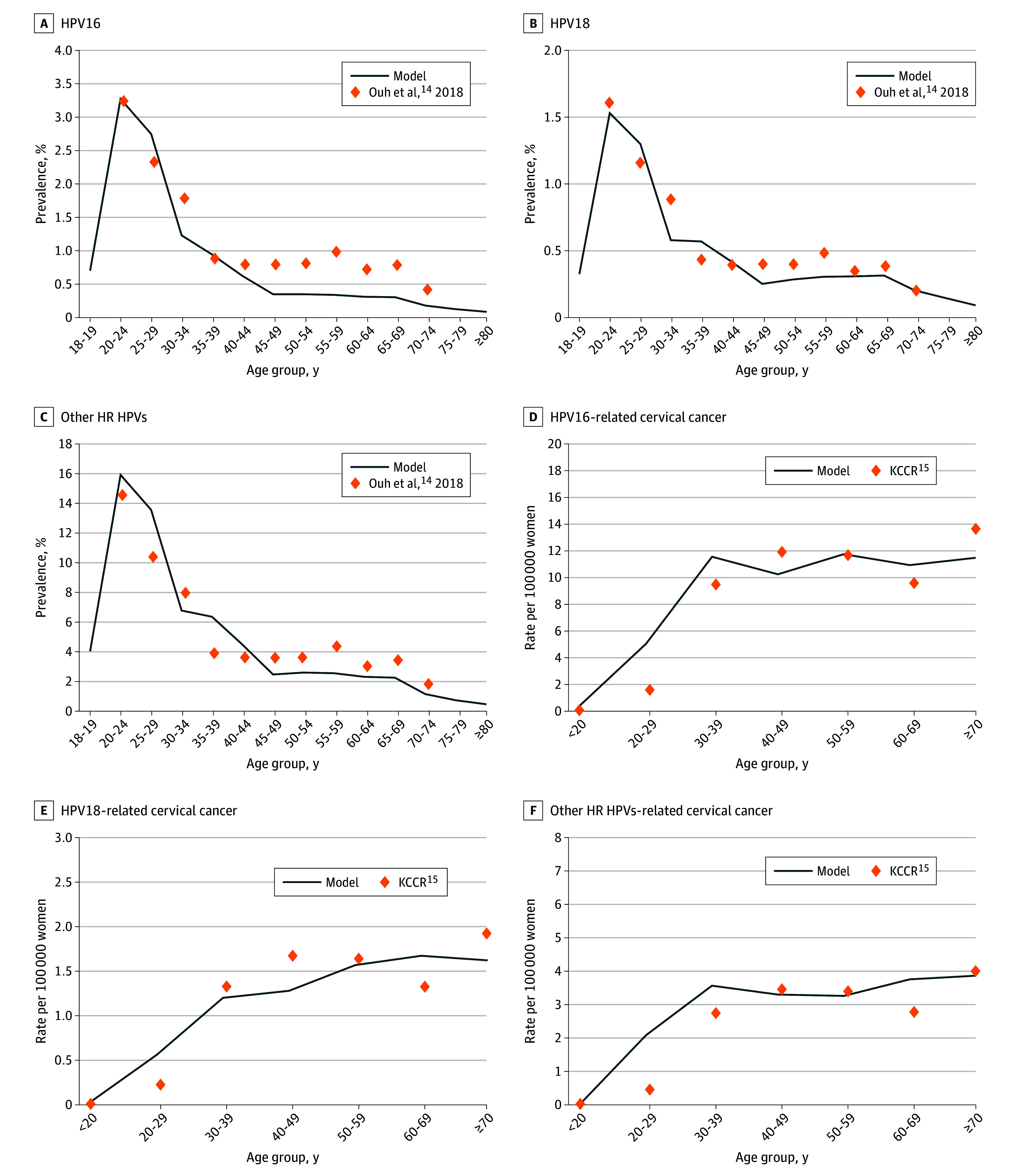
Age-Specific Prevalence of Human Papillomavirus (HPV) and Cervical Cancer, Projected By Model and Epidemiological Data HR indicates high risk; KCCR, Korea Center Cancer Registry.

The model-projected population of more than 50 million Koreans, approximately 50% of whom are women, is illustrated in eFigure 2 in Supplement 1. From 2010 (n = 49 554 112) to 2020 (n = 51 836 239), the model captured a slight increase in both the total population size and the female population. After 2020, the population was estimated to steadily decline to approximately 35 million by 2070 and fewer than 30 million by 2100. The projected population size and trends closely aligned with the demographic data reported by the Korean Statistical Information Service.^22,23^ The model successfully captured the age-specific prevalence of HPV infection and the incidence of cervical cancer (Figure 1).

### Cervical Cancer Incidence and Deaths

The projected cumulative cervical cancer cases and deaths in 2100 for the selected strategies under realistic and ideal scenarios are presented in Table 1. Among all Pap testing–based approaches, the current strategy (biennial screening starting at age 20 years with no upper age limit) was the most effective in preventing invasive cervical cancer, resulting in 47 358 cases by 2100 in the realistic scenario (Table 1; eTable 5 in Supplement 1). Notably, 2-year hrHPV screening strategies, except for those with an upper age limit of 64 years, prevented approximately 20% to 27% more cervical cancer cases compared with the current Pap test–based screening. Regarding cervical cancer deaths, the current screening policy was estimated to result in the lowest number of deaths among all Pap testing–based screening strategies. Extending the Pap testing screening interval to 5 years was estimated to lead to approximately 1.4 times more cervical cancer deaths by 2100 (Table 1; eTable 6 in Supplement 1). In contrast, hrHPV strategies, including biennial strategies starting at age 20 years or 25 years with either no upper age limit or ending at age 74 years, were estimated to reduce cervical cancer deaths by 3700 (−11%) to 4700 (−14%) compared with the current Pap testing–based screening policy.

**Table 1.** Projected Cumulative Cervical Cancer Cases and Deaths by 2100 Under Realistic and Ideal Scenarios

Interval, y	Starting age, y	Ending age, y	Cervical cancer cases, No.				Cervical cancer death, No.				
			Realistic scenarios^a^		Ideal scenarios^b^		Realistic scenarios^a^		Ideal scenarios^b^	
			Cases	Adverted cases	Cases	Adverted cases	Deaths	Adverted cases	Deaths	Adverted cases
**Papanicolaou test–based strategies**											
2	20	NA	47 358	0 [Reference]	39 522	0 [Reference]	34 242	0 [Reference]	31 149	0 [Reference]
2	25	NA	47 906	−548	40 373	−851	34 555	−313	31 635	−487
2	25	64	57 915	−10 557	53 119	−13 597	38 007	−3765	35 763	−4614
2	25	74	49 960	−2602	44 387	−4865	35 148	−906	32 692	−1543
3	25	NA	59 202	−11 844	50 356	−10 834	39 718	−5476	36 047	−4898
3	25	64	67 884	−20 526	61 949	−22 427	42 804	−8562	39 916	−8767
3	25	74	60 903	−13 545	54 100	−14 578	40 221	−5979	37 070	−5921
5	25	NA	74 620	−27 262	64 872	−25 350	47 065	−12 823	42 785	−11 636
5	25	64	81 585	−34 227	74 746	−35 224	49 616	−15 374	46 188	−15 039
5	25	74	75 920	−28 562	68 160	−28 638	47 459	−13 217	43 720	−12 571
**hrHPV testing–based strategies**											
2	25	NA	35 302	12 056	29 750	9772	29 887	4354	27 855	3294
2	25	64	46 966	392	43 729	−4207	33 583	659	32 080	−932
2	25	74	37 868	9490	34 020	5502	30 547	3695	28 888	2261
3	25	NA	44 426	2932	37 391	2131	33 957	285	31 186	−37
3	25	64	54 868	−7510	50 481	−10 959	37 340	−3098	35 221	−4072
3	25	74	46 605	753	41 480	−1958	34 528	−286	32 199	−1050
5	25	NA	58 281	−10 923	49 523	−10 001	40 402	−6160	36 725	−5576
5	25	64	67 068	−19 710	61 213	−21 691	43 315	−9073	40 405	−9256
5	25	74	60 008	−12 650	53 291	−13 769	40 863	−6621	37 685	−6536

Abbreviations: hrHPV, high-risk human papillomavirus; NA, not applicable.

^a^ The realistic scenario uses the age-specific screening rate of 51.5% from the National Cancer Screening Program and the HPV vaccination rate of 75% in adolescent girls.^13^

^b^ The ideal scenario uses the realistic scenario from 2024 to 2030, then from 2030, the screening rate increases to 70% and the HPV vaccination rate increases to 90% in adolescent girls.

On average, the ideal scenario resulted in approximately 6500 (12%) fewer cervical cancer cases than the realistic scenario. The reduction varied from 7% (for biennial hrHPV testing from age 25 to 64 years) to 17% (for biennial Pap testing from age 20 years with no upper age limit and Pap testing every 5 years from age 25 to 74 years), depending on the screening strategy (Table 1; eTable 7 in Supplement 1). For cervical cancer deaths, the ideal scenario prevented an average of 3000 (7%) more cervical cancer deaths than the realistic scenario (Table 1; eTable 8 in Supplement 1).

### Timeframe to Cervical Cancer Elimination

**Figure 2.**
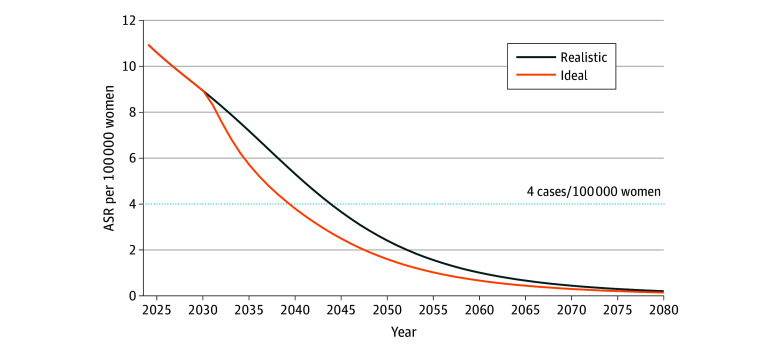
Projected Timeframe to Cervical Cancer Elimination Under Current Screening Strategies in Korea The realistic scenario uses the age-specific screening rate of 51.5% from the of the National Cancer Screening Program and the human papillomavirus (HPV) vaccination rate of 75% in adolescent girls.^13^ The ideal scenario uses the realistic scenario from 2024 to 2030, then from 2023, the screening rate increases to 70% and the HPV vaccination rate increases to 90% in adolescent girls. ASR indicates age-standardized rate.

Under the realistic scenario, reflecting current screening and vaccination rates, incidence was estimated to fall below the WHO elimination threshold by 2044 with the current screening strategy (Figure 2). Starting screening at age 25 years and/or setting an upper age limit of 74 years was estimated to have a similar elimination timeline under the same Pap test screening interval (Table 2; eFigure 3 in Supplement 1). However, extending the Pap screening interval to 5 years could delay elimination by 10 years or more (eFigure 4 in Supplement 1). Regarding the screening test, 2-year hrHPV screening strategies (eg, starting at ages 20 or 25 years with no upper age limit or starting at age 20 years and ending at age 74 years) could accelerate cervical cancer elimination, reaching the threshold as early as 2038 in the realistic scenario. In contrast, 3-year hrHPV screening showed a similar elimination timeline to the current policy, while 5-year hrHPV testing delayed elimination by up to 6 years (Table 2; eFigure 5 in Supplement 1). Under the ideal scenario, with 70% screening and 90% vaccination coverage from 2030, the current policy could achieve cervical cancer elimination by 2040. In contrast, adopting 2-year hrHPV testing could accelerate elimination to 2034.

**Table 2.** Projected Year of Cervical Cancer Elimination in South Korea

Interval, y	Starting age, y	Ending age, y	Realistic scenarios^a^				Ideal scenarios^b^			
			ASR of cervical cancer per 100 000 women			Year of elimination^c^	ASR of cervical cancer per 100 000 women			Year of elimination^c^
			2030	2040	2050		2030	2040	2050	
**Papanicolaou testing–based strategy**										
2	20	NA	9.0	5.3	2.4	2044	9.0	3.8	1.6	2040
2	25	NA	9.0	5.4	2.5	2045	9.0	4.0	1.7	2040
2	25	64	9.7	6.3	3.0	2047	9.7	5.1	2.4	2044
2	25	74	9.2	5.6	2.6	2045	9.2	4.3	1.9	2041
3	25	NA	10.6	7.4	3.6	2049	10.6	5.7	2.5	2045
3	25	64	11.1	8.1	4.1	2051	11.1	6.8	3.2	2048
3	25	74	10.7	7.5	3.7	2049	10.7	6.0	2.7	2046
5	25	NA	12.2	10.0	5.4	2054	12.2	8.4	4.1	2051
5	25	64	12.5	10.5	5.8	2055	12.5	9.2	4.7	2053
5	25	74	12.2	10.1	5.4	2054	12.2	8.6	4.2	2051
**hrHPV testing–based strategy**										
2	25	NA	6.7	3.4	1.5	2038	6.7	2.4	1.0	2034
2	25	64	7.6	4.4	2.1	2042	7.6	3.6	1.7	2039
2	25	74	6.9	3.6	1.6	2039	6.9	2.7	1.2	2036
3	25	NA	8.5	4.8	2.2	2043	8.5	3.5	1.5	2039
3	25	64	9.2	5.7	2.8	2046	9.2	4.7	2.2	2043
3	25	74	8.6	5.0	2.3	2043	8.6	3.8	1.7	2040
5	25	NA	10.5	7.2	3.5	2049	10.5	5.6	2.5	2045
5	25	64	11.0	7.9	4.0	2050	11.0	6.6	3.1	2047
5	25	74	10.6	7.3	3.6	2049	10.6	5.9	2.6	2046

Abbreviations: ASR, age-standardized rate; hrHPV, high-risk human papillomavirus.

^a^ The realistic scenario uses the age-specific screening rate of 51.5% from the of the National Cancer Screening Program and the HPV vaccination rate of 75% in adolescent girls.^13^

^b^ The ideal scenario uses the realistic scenario from 2024 to 2030, then from 2030, the screening rate increases to 70% and the HPV vaccination rate increases to 90% in adolescent girls.

^c^ ASR ≤4 cases per 100 000 women.

### Sensitivity Analysis

**Figure 3.**
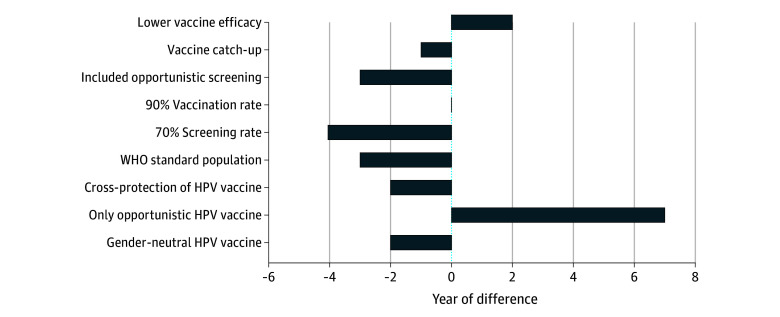
Difference in Time to Cervical Cancer Elimination Under Current Screening Strategies in Realistic Scenarios With Different Parameters Current strategy is biennial Papanicolaou testing for women aged 20 years or older; the projected year of elimination is 2044. HPV indicates human papillomavirus; WHO, World Health Organization.

Sensitivity analysis highlighted that opportunistic vaccination alone could delay cervical cancer elimination by up to 7 years (Figure 3). Assuming a lower vaccine efficacy of 80% would delay elimination by 2 years. Using the WHO World Standard Population advanced the elimination timeline by 3 to 4 years. In contrast, sex-neutral vaccination, catch-up vaccination to age 25 years, or cross-protection of the HPV vaccine had minimal impact on the estimated elimination year. Notably, under current screening and vaccination rates, opportunistic screening could lead to elimination by 2041.

## Discussion

Under a realistic scenario reflecting Korea’s current HPV vaccination and screening rates, our model found that the current strategy—a biennial Pap test for women aged 20 years or older—was the most effective among the Pap testing–based options. Adjusting the starting age to 25 years or adjusting the upper age limit to 74 years yielded similar outcomes. In contrast, all 2-year hrHPV screening strategies consistently outperformed Pap test strategies. In a realistic scenario, cervical cancer was projected to be eliminated by 2044 under current policies, while switching to 2-year hrHPV screening could accelerate elimination to as early as 2038. In an ideal scenario—achieving 90% vaccination and 70% screening coverage from 2030—all strategies further reduced the cancer burden, preventing 12% more cases and 7% more deaths compared with the realistic scenario. Particularly, hrHPV with a 2-year interval was estimated to lead to early elimination by 2034.

These findings underscore that shifting to HPV-based screening has a greater impact on reducing cervical cancer incidence and mortality than merely increasing screening and HPV vaccination rates. This suggests that adopting a more effective screening strategy may offer greater benefits than achieving ideal coverage with a suboptimal test. However, transitioning to HPV testing involves procedural and financial challenges. Therefore, a dual strategy, gradually shifting toward HPV-based screening while continuing to improve participation rates, may offer the most effective path toward achieving the WHO cervical cancer elimination target.

Sensitivity analysis showed that achieving a 70% screening rate by 2030 could advance cervical cancer elimination by 2040 under current policies. Similarly, increasing the vaccination rate to 90% by 2030 projected a cervical cancer elimination year of 2044. The greater impact of screening over vaccination on the elimination timeline aligns with previous studies.^24,25^ This is largely due to the nature of prevention: HPV vaccination, administered to girls aged 12 to 15 years before sexual activity, takes decades to affect cervical cancer incidence, which typically occurs in women older than 30 years. In contrast, screening detects and removes precancerous lesions, leading to more immediate reductions in incidence. Thus, it is reasonable to conclude that cervical cancer screening would have a more immediate effect on accelerating elimination.

Although HPV vaccination was officially introduced in the NIP in 2016, its full impact has yet to be realized. To assess its importance, we conducted a sensitivity analysis assuming only opportunistic vaccination. The results highlight the critical role of government-led vaccination programs in reducing the cervical cancer burden. Without the national HPV vaccination program, elimination could be delayed by up to 7 years. These findings also offer valuable implications for countries with similar disease burdens, emphasizing the need to expand HPV vaccination efforts to meet elimination goals. While our analysis was limited to 2-valent and 4-valent vaccines, previous studies have shown that the 9-valent HPV vaccine offers superior effectiveness and cost-effectiveness by covering additional high-risk HPV genotypes (31, 33, 45, 52, and 58). Transitioning from the 4-valent to the 9-valent vaccine could therefore lead to greater reductions in HPV prevalence and cervical cancer incidence.^26,27^

Previous global estimates using the Policy1-Cervix simulation platform projected that South Korea would reach the cervical cancer elimination threshold by 2060 to 2065,^28^ nearly 2 decades later than our model’s projections. This discrepancy largely stems from key differences in model structure and assumptions. Policy1-Cervix is an individual-based microsimulation model that simulates disease progression stochastically at the individual level. In contrast, our deterministic, age-structured, dynamic model estimates population-level outcomes. In addition, the global estimates did not account for South Korea’s unique context, including the implementation of its NIP and existing levels of opportunistic HPV vaccination. Screening assumptions also differed: previous estimates used screening once or twice in a lifetime, while our model incorporated routine 2-, 3-, and 5-year screening intervals. Most notably, they assumed a delayed start (2020) and lower HPV vaccine uptake (50%), far below actual coverage in South Korea.

In Australia, Hall et al^29^ used the Policy1-Cervix model to estimate that the country could reach 6 cervical cancer cases per 100 000 women by 2020 (2018-2022) and achieve full elimination by 2028, depending on the scenario. This accelerated timeline reflects Australia’s early introduction of HPV vaccination in 2012 through its NIP, which included both girls and boys, with high coverage rates of 83.2% and 75.5%, respectively.^29^ In the United States, a comparative modeling study using 2 CISNET-Cervical microsimulation models (Harvard and Policy1-Cervix)^24^ projected elimination between 2038 and 2046 under current vaccination and screening practices. With screening coverage scaled up to 90%, incidence could fall below 4 cases per 100 000 women as early as 2028 (Harvard model) or by 2033 (Policy1-Cervix model).^24^ Notably, the models found no significant difference in the elimination timeline between girls-only and sex-neutral vaccination strategies, consistent with our sensitivity analysis. The earlier projected elimination in the United States is also attributed to its relatively low baseline cervical cancer burden (ASR of approximately 7 per 100 000 women in 2019).^24^ Malaysia had a cervical cancer incidence of approximately 10 cases per 100 000 women in 2020 and is projected to achieve elimination between 2056 and 2062 through HPV vaccination and twice-in-a-lifetime screening for women aged 35 to 45 years.^30^ This is approximately 2 decades later than South Korea, mainly due to less frequent screening and the delayed implementation of the HPV vaccination program.

### Limitations

This study has several limitations. First, our deterministic model provides population-level average estimates but does not account for individual-level stochastic variations, which may be relevant for smaller subgroups, such as women with hysterectomy, those living with HIV, or individuals affected by socioeconomic disparities. For instance, progression and regression rates between precancerous stages were based on national averages from the KCCR and do not reflect potentially faster disease progression in immunocompromised populations, such as women living with HIV. These groups may experience different disease trajectories, and their exclusion represents a recognized limitation. Nevertheless, our modeling approach is well-suited for long-term projections using aggregated national data and is computationally efficient, enabling us to explore multiple scenarios and conduct sensitivity analyses. Second, our model is sensitive to initial parameter values. While we conducted 1-way sensitivity analyses to evaluate the impact of individual parameters, this approach does not capture joint uncertainty across multiple parameters. Incorporating probabilistic sensitivity analyses in future research could enhance the robustness of uncertainty estimates. Third, our model assumed independent transmission of HPV genotypes and simulated each genotype-specific natural history in separate submodels. As a result, co-infections—simultaneous infections with multiple HPV types—were not explicitly modeled. This approach does not account for potential biological interactions between HPV types, such as competitive inhibition or synergistic effects on viral clearance and disease progression. This simplification might influence the precise dynamics of natural history transitions and, subsequently, the overall burden estimates, particularly in settings where co-infection is common. In addition, due to limited data, our model did not include all HPV genotypes and could not account for 100% of cervical cancer cases. However, the 9 high-risk HPV genotypes included in the model successfully captured the pattern of age-specific incidence patterns and accounted for approximately 94% of cervical cancer cases. Therefore, the model’s projections closely reflect real-world trends. As more data on rare genotypes become available, future models may incorporate them to improve precision. Finally, this study focused on projecting cervical cancer cases and deaths to estimate the year of elimination, without incorporating economic evaluation. Future cost-effectiveness analyses would be valuable to inform decisions on the most efficient and feasible screening and vaccination strategies.

## Conclusions

The findings of this modeling study showed that under current screening and vaccination policies, South Korea could eliminate cervical cancer by 2044. Transitioning to hrHPV testing could improve outcomes, preventing up to 27% more cases and advancing elimination to 2038. Under an ideal scenario with 70% screening and 90% vaccination coverage, 2-year hrHPV testing could achieve elimination as early as 2034. These findings underscore the importance of combining high coverage with effective screening strategies to accelerate elimination and meet WHO targets.

## Notes

Supplement 1. **eAppendix.** Supplemental Methods **eTable 1.** Key Model Assumptions **eTable 2.** Screening Strategies to Evaluate in the Study **eTable 3.** Key Scenarios and Assumptions for the Sensitivity Analysis **eTable 4.** List of Input Parameters for the Model **eTable 5.** Cumulative Cervical Cancer Cases From 2024 to 2100 According to Screening Strategies in Realistic Scenarios **eTable 6.** Cumulative Cervical Cancer Deaths From 2024 to 2100 According to Screening Strategies in Realistic Scenarios **eTable 7.** Projected Total Cervical Cancer Cases and Cases Averted by 2100 **eTable 8.** Projected Total Cervical Cancer Deaths and Deaths Averted by 2100 **eFigure 1.** Simplified Structure of the Model **eFigure 2.** Population Size Over the Projected Period Compared With KOSIS Data and Projection **eFigure 3.** Trend In Age-Standardized Cervical Cancer Incidence According to Screening Strategies Toward Cervical Cancer Elimination Under Realistic Scenarios **eFigure 4.** Trend In Age-Standardized Cervical Cancer Incidence According to Screening Strategies Using Pap Smear Under Realistic Scenarios **eFigure 5.** Trend In Age-Standardized Cervical Cancer Incidence According to Screening Strategies Using HPV Testing Under Realistic Scenarios
eReferences.

Supplement 2.
Data Sharing Statement
