# Supplementary material for: Cervical Cancer Screening, HPV Vaccination, and Cervical Cancer Elimination
Source: JAMA Netw Open. 2025 Aug 12;8(8):e2526683. doi: 10.1001/jamanetworkopen.2025.26683 (PMC12344532; doi:10.1001/jamanetworkopen.2025.26683)
Supplement: Supplement 1. — eAppendix. Supplemental Methods eTable 1. Key Model Assumptions eTable 2. Screening Strategies to Evaluate in the Study eTable 3. Key Scenarios and Assumptions for the Sensitivity Analysis eTable 4. List of Input Parameters for the Model eTable 5. Cumulative Cervical Cancer Cases From 2024 to 2100 According to Screening Strategies in Realistic Scenarios eTable 6. Cumulative Cervical Cancer Deaths From 2024 to 2100 According to Screening Strategies in Realistic Scenarios eTable 7. Projected Total Cervical Cancer Cases and Cases Averted by 2100 eTable 8. Projected Total Cervical Cancer Deaths and Deaths Averted by 2100 eFigure 1. Simplified Structure of the Model eFigure 2. Population Size Over the Projected Period Compared With KOSIS Data and Projection eFigure 3. Trend In Age-Standardized Cervical Cancer Incidence According to Screening Strategies Toward Cervical Cancer Elimination Under Realistic Scenarios eFigure 4. Trend In Age-Standardized Cervical Cancer Incidence According to Screening Strategies Using Pap Smear Under Realistic Scenarios eFigure 5. Trend In Age-Standardized Cervical Cancer Incidence According to Screening Strategies Using HPV Testing Under Realistic Scenarios eReferences. [file jamanetwopen-e2526683-s001.pdf]

Supplemental Online Content

Luu XQ, Jun JK, Suh M, Oh J, Yu S, Choi KS. Cervical cancer screening, HPV vaccination, and cervical cancer elimination. *JAMA Netw Open.* 2025;8(8):e2526683. doi:10.1001/jamanetworkopen.2025.26683

eAppendix. Supplemental Methods

eTable 1. Key Model Assumptions

eTable 2. Screening Strategies to Evaluate in the Study

eTable 3. Key Scenarios and Assumptions for the Sensitivity Analysis

eTable 4. List of Input Parameters for the Model

eTable 5. Cumulative Cervical Cancer Cases From 2024 to 2100 According to Screening Strategies in Realistic Scenarios

eTable 6. Cumulative Cervical Cancer Deaths From 2024 to 2100 According to Screening Strategies in Realistic Scenarios

eTable 7. Projected Total Cervical Cancer Cases and Cases Averted by 2100

eTable 8. Projected Total Cervical Cancer Deaths and Deaths Averted by 2100

eFigure 1. Simplified Structure of the Model

eFigure 2. Population Size Over the Projected Period Compared With KOSIS Data and Projection

eFigure 3. Trend In Age-Standardized Cervical Cancer Incidence According to Screening Strategies Toward Cervical Cancer Elimination Under Realistic Scenarios

eFigure 4. Trend In Age-Standardized Cervical Cancer Incidence According to Screening Strategies Using Pap Smear Under Realistic Scenarios

eFigure 5. Trend In Age-Standardized Cervical Cancer Incidence According to Screening Strategies Using HPV Testing Under Realistic Scenarios

eReferences.

This supplemental material has been provided by the authors to give readers additional information about their work.

© 2025 Luu XQ et al. *JAMA Network Open*.

eAppendix. Supplemental Methods

Model equation

For every compartment X[a,s](t) at time t, the compartment has a transition rate of Δ(X[a,s](t)), which is the combination of the birth rate for the youngest age group or aging rate for the other groups, and the age-specific death rate excluding cervical cancer death m[a,s].

Force of infection

The force of infection at time (t) is defined as a function of the transmission rate (β, $\bar{\beta}$), sexual behavior of the population (probability of forming a new sexual partnership: ψ[a] $\bar{\psi}$[a]; effective partnership change rate: $\bar{\kappa}$[a] κ[a]), and proportion of HPV infection in the population at time (t).

Female

$\lambda[a](t) = \beta * \psi[a] * \kappa[a] * \sum_{i=2}^{12} \chi ai * \frac{I[i,m](t)}{N[i,m](t)}$

Male

$\bar{\lambda}[a](t) = \bar{\beta} * \bar{\psi}[a] * \bar{\kappa}[a] * \sum_{i=2}^{12} \bar{\chi} ai * \frac{I[i,f](t)}{N[i,f](t)}$

The effective partnership change ($\bar{\kappa}$[a] κ[a]) is the average number of new partners per year, which is calculated using the following equation:

$\kappa[a] = \kappa \sum_k \kappa\, Pr_\lambda(\kappa) = \sum_k \kappa * [Pr_\pi(\kappa)] / [\sum_k \kappa * Pr_\pi(\kappa)] = E_\pi(\kappa^2) / E_\pi(\kappa) = m_\pi + \sigma_\pi^2 / m_\pi$

where $Pr_\pi(\kappa)$ and $Pr_\lambda(\kappa)$ are the distribution of κ in the general population and STD population, respectively; m_π is the average number of new partners in the prior year in the general population, and σ_π^2 represents the variance.

Transmission of HPV

Male

$\frac{dS[a]}{dt} = -\bar{\lambda}[a](t)*\bar{S}[a] + (1-\alpha)*\gamma*\bar{I}[a] + \Delta(\bar{S}[a](t))$

$\frac{dI[a]}{dt} = \bar{\lambda}[a](t)*(\bar{S}[a] + \bar{R}[a] + \bar{V}[a]) + \Delta(\bar{I}[a](t))$

$\frac{dR[a]}{dt} = \alpha*\gamma*\bar{I}[a] - 0.7*\bar{\lambda}[a](t)*\bar{R}[a] + \Delta(\bar{R}[a](t))$

$\frac{dV[a]}{dt} = -\bar{\lambda}[a](t)*(1-\eta)*\bar{V}[a] + \Delta(\bar{V}[a](t))$

Female

$\frac{dS[a]}{dt} = -\lambda[a](t)*S[a] + (1-\alpha)*\gamma*I[a] + (\rho_{CIN1\text{-}SI}/2)*CIN1[a] + p[a]*se*CIN23[a] + \Delta(S[a](t))$

$\frac{dI[a]}{dt} = \lambda[a](t)*(S[a] + (1-\eta)*V[a] + 0.7*R[a]) - \gamma*I[a] - (\rho_{I\text{-}CIN1})*I[a] + (\rho_{CIN1\text{-}SI}/2)*CIN1[a] + \Delta(I[a](t))$

$\frac{dR[a]}{dt} = \alpha*\gamma*I[a] - 0.7*\lambda[a](t)*R[a] + \Delta(R[a](t))$

$\frac{dV[a]}{dt} = -(1-\eta)*\lambda[a](t)*V[a] + \Delta(V[a](t))$

Disease stages

$\frac{dCIN1[a]}{dt} = \rho_{I\text{-}CIN1}*I[a] + \rho_{CIN23\text{-}1}*CIN23[a] - \rho_{CIN1\text{-}SI}*CIN1[a] - \rho_{CIN1\text{-}23}*CIN1[a] + \Delta(CIN1[a](t))$

$\frac{dCIN23[a]}{dt} = \rho_{CIN1\text{-}23}*CIN1[a] - \rho_{CIN23\text{-}1}*CIN23[a] - p*se*CIN23[a] - \rho_{CIN3\text{-}C}*CIN23[a] + \Delta(CIN23[a](t))$

$\frac{dLCC[a]}{dt} = \rho_{CIN3\text{-}C}*CIN23[a] - \rho_{LCC\text{-}RCC}*LCC[a] - p*se*LCC[a] - sym_L*LCC[a] - \mu_L*LCC[a] - \Delta(LCC[a])$

$\frac{dDLCC[a]}{dt} = p*se*LCC[a] + sym_L*LCC[a] - \mu_L*DLCC[a] - \Delta(DLCC[a])$

$\frac{dRCC[a]}{dt} = \rho_{LCC\text{-}RCC}*LCC[a] - p[a]*se*RCC[a] - sym_R*RCC[a] - \mu_R*RCC[a] + \Delta(RCC[a])$

$\frac{dDRCC[a]}{dt} = p[a]*see*RCC[a] + sym_R*RCC[a] - \mu_R*DRCC[a] - \Delta(DRCC[a])$

$\frac{dDCC[a]}{dt} = \rho_{LCC\text{-}RCC}*RCC[a] - \mu_D*DCC[a] - \Delta(DCC[a])$

Note: *The detailed explanation of all symbols in model equations is provided in eTable 4.*

eTable 1. Key Model Assumptions

**Modules**	**Assumptions**
Population demographic	• No entry or exit due to migration.<br>• The age of sexual debut is 18 years
HPV infection and vaccination	• The infection of different HPV genotypes is independent
Nature history of cervical cancer	• Invasive cervical cancer is only developed from HPV infected women
Screening	• The follow-up rate of positive screening results is 90%<br>• All screening detected CIN2/3 is treated successfully<br>• The detected/diagnosed invasive cancer cases remain in the stage of detection/diagnosis

© 2025 Luu XQ et al. *JAMA Network Open*.

eTable 2. Screening Strategies to Evaluate in the Study

**Strategy characteristic**	**Considered values**
Primary screening test	Cytology (Pap smear)<br>hrHPV testing
Age of screening initiation	20, 25
Age of screening cessation	65, 75, non
Screening interval (years)	2, 3, 5

© 2025 Luu XQ et al. *JAMA Network Open*.

eTable 3. Key Scenarios and Assumptions for the Sensitivity Analysis

Scenarios	Assumptions
The absence of HPV vaccine in NIP	There was only opportunistic HPV vaccination among girls with a vaccination rate of 20%.<br>*The optimistic assumption of an opportunistic vaccination rate was made based on results of a national survey in 2016, wherein 16.9% of girls aged 9–19 years received at least 1-2 dose of vaccination (1)*
Gender-neutral HPV vaccination program	Both boys and girls were included in NIP for HPV vaccination with a vaccination rate of 75%
Cross-protection of HPV vaccine	The 4-valent vaccine was assumed to protect people against some other HR HPVs including HPV31/*33/45/52/58.*<br>*Based on the previous study the effectiveness of the vaccine on other HR genotypes was 0.46 for HPV31, 0.29 for HPV33, 0.08 for HPV45, 0.18 for HPV52, and 0.06 for HPV58 (2).*
The consideration of opportunistic screening rate	Beside of 2019 age-specific screening rate in NCSP, the model consider the age-specific opportunistic screening rate (overall rate of 9.0%) using results of National Cancer Screening Survey 2019.
70% of screening rate	While the vaccination rate remained at 75%, the country could reach the WHO target on screening rate of 70%
90% of vaccination rate	While the screening rate remained at 2019 age-specific screening rate, the country could reach the WHO target on vaccination rate of 90%
WHO World Standard Population	Variation in age structure was accounted for by assuming the WHO World Standard population (3).
Lower vaccine efficacy	Vaccine efficacy of 80%
Vaccine catch-up	HPV vaccination catchup to 25 year olds

© 2025 Luu XQ et al. *JAMA Network Open*.

eTable 4. List of Input Parameters for the Model

**Parameter description**	**Symbol**	**Sources**	**Reference**
Age and sex distribution of the population		KOSIS	(4)
Fertility rate	b	KOSIS	(5)
Age-specific death rate excluding cervical cancer death	m[a,s]	KOSIS	(6)
Probability among women of age *a* of forming a new sexual partnership	ψ[a]	Korean HPV infection-related sexual behavior survey, 2022	(2)
Probability among men of age *a* of forming a new sexual partnership	$\bar{\psi}$[a]	Korean HPV infection-related sexual behavior survey, 2022	(2)
Effective partnership change rate among women of age *a*	κ[a]	Korean HPV infection-related sexual behavior survey, 2022	(2)
Effective partnership change rate among men of age *a*	$\bar{\kappa}$[a]	Korean HPV infection-related sexual behavior survey, 2022	(2)
Age-distribution of female partners of age *i* for a male of age *a*	χ_{ai}	Korean HPV infection-related sexual behavior survey, 2022	(2)
Age-distribution of male partners of age i for a female of age a	$\bar{\chi}_{ai}$	Korean HPV infection-related sexual behavior survey, 2022	(2)
**Epidemiology of HPV**			
Proportion of infections that develop HPV-type specific antibodies	α	0.5	Assumption
Force infection	λ	Calculation	
Clearance rate in women	γ	Calibration	
Clearance rate in men	$\bar{\gamma}$	Calibration	
Per partner rate of female to male transmission	β	Calibration	
Per partner rate of male to female transmission	$\bar{\beta}$	Calibration	
Relative risk of reinfection after clearance		0.7	Assumption
**Natural history of cervical cancer**			
Rate of HPV infection progressing to CIN1	$\rho_{I\text{-}CIN1}$	Calibration	
Rate of CIN1 progressing to CIN2/3	$\rho_{1\text{-}CIN23}$	Calibration	
Rate of CIN2/3 progressing to Invasive cancer	$\rho_{CIN23\text{-}C}$	Calibration	
Rate of CIN1 regression to normal cytology/infection (Assuming 50% of CIN1 cases regress to HPV infection)	$\rho_{CIN1\text{-}SI}$	Calibration	
Rate of CIN2/3 regressing to CIN1	$\rho_{CIN23\text{-}1}$	Calibration	
Rate of LCC progressing to RCC	$\rho_{LCC\text{-}RCC}$	22% yr-1	(7, 8)
Rate of RCC progressing to DCC	$\rho_{RCC\text{-}DCC}$	26% yr-1	(7, 8)

© 2025 Luu XQ et al. *JAMA Network Open*.

Parameter description	Symbol	Sources	Reference
Mortality rate of cervical cancer	μ_L<br>μ_R<br>μ_D		(9)
**Cervical cancer screening**			
Age specific rates of screening	p	Korean National Cancer Screening Program 2019	
Stage-specific probability of diagnosing cervical cancer/symptom detection (without screening)	sym_L<br>sym_R<br>sym_D	LCC: 0.21 yr-1<br>RCC: 0.961 yr-1<br>DCC: 2.30 yr-1	(7, 8)
Sensitivity of screening for CIN2+	se	Pap test<br>CIN2/3:<br>0.55 (0.34-0.77)<br>Invasive cancer: 0.867	(10, 11)
		hrHPV<br>0.94 (0.84- 1.00)	(10)
Specificity of screening for CIN2+	spe	**PAP test**<br>CIN2/3:<br>0.97 (0.96-0.97)<br>Invasive cancer: 0.98	(10, 11)
		**hrHPV**<br>0.94 (0.93- 0.95)	(10)
**Seroprevalence**			
Type-specific HPV prevalence			(12, 13)
**Vaccination**			
Effectiveness of HPV vaccine on HPV16/18	η	0.9	Assumption
Vaccination rate (girls)	v	0.75	(14)

© 2025 Luu XQ et al. *JAMA Network Open*.

eTable 5. Cumulative Cervical Cancer Cases From 2024 to 2100 According to Screening Strategies in Realistic Scenarios

Strategies[a]	2025	2030	2035	2040	2045	2050	2060	2070	2080	2090	2100	Diff[b] (No.)	Diff[b] (%)
1_PAP_2_20_non	5,521	17,555	27,092	34,134	38,978	42,138	45,369	46,604	47,082	47,276	47,358	Ref	Ref
2_PAP_2_20_64	5,521	18,282	29,427	38,256	44,738	49,244	54,202	56,198	56,956	57,242	57,354	9,996	21.1
3_PAP_2_20_74	5,521	17,721	27,602	35,005	40,170	43,593	47,171	48,566	49,106	49,321	49,410	2,052	4.3
4_PAP_2_25_non	5,521	17,599	27,236	34,387	39,323	42,551	45,862	47,130	47,622	47,821	47,906	548	1.2
5_PAP_2_25_64	5,521	18,325	29,571	38,509	45,083	49,659	54,699	56,732	57,506	57,799	57,915	10,557	22.3
6_PAP_2_25_74	5,521	17,764	27,745	35,257	40,514	44,006	47,664	49,094	49,647	49,868	49,960	2,602	5.5
7_PAP_3_20_non	5,521	18,717	30,593	39,962	46,682	51,187	55,875	57,658	58,332	58,597	58,708	11,350	24.0
8_PAP_3_20_64	5,521	19,240	32,365	43,207	51,341	57,053	63,365	65,909	66,874	67,238	67,380	20,022	42.3
9_PAP_3_20_74	5,521	18,832	30,963	40,616	47,600	52,332	57,331	59,268	60,003	60,290	60,407	13,049	27.6
10_PAP_3_25_non	5,521	18,747	30,698	40,156	46,959	51,532	56,306	58,128	58,817	59,089	59,202	11,844	25.0
11_PAP_3_25_64	5,521	19,270	32,470	43,401	51,618	57,398	63,799	66,384	67,368	67,739	67,884	20,526	43.3
12_PAP_3_25_74	5,521	18,862	31,068	40,810	47,878	52,677	57,762	59,738	60,489	60,782	60,903	13,545	28.6
13_PAP_5_20_non	5,521	19,811	34,279	46,605	55,983	62,578	69,768	72,589	73,644	74,043	74,202	26,845	56.7
14_PAP_5_20_64	5,521	20,146	35,472	48,876	59,347	66,923	75,534	79,091	80,454	80,964	81,160	33,802	71.4
15_PAP_5_20_74	5,521	19,883	34,519	47,043	56,616	63,385	70,834	73,796	74,912	75,335	75,501	28,143	59.4
16_PAP_5_25_non	5,521	19,829	34,347	46,738	56,183	62,837	70,112	72,977	74,051	74,458	74,620	27,262	57.6
17_PAP_5_25_64	5,521	20,165	35,540	49,009	59,547	67,183	75,880	79,482	80,866	81,386	81,585	34,227	72.3
18_PAP_5_25_74	5,521	19,901	34,587	47,176	56,816	63,645	71,178	74,184	75,319	75,750	75,920	28,562	60.3
19_HPV_2_20_non	5,521	15,633	22,115	26,577	29,549	31,462	33,426	34,194	34,500	34,627	34,681	**-12,677**	**-26.8**
20_HPV_2_20_64	5,521	16,707	25,284	31,894	36,737	40,130	43,911	45,447	46,027	46,245	46,329	**-1,029**	**-2.2**

© 2025 Luu XQ et al. *JAMA Network Open*.

Strategies[a]	2025	2030	2035	2040	2045	2050	2060	2070	2080	2090	2100	Diff[b] (No.)	Diff[b] (%)
21_HPV_2_20_74	5,521	15,893	22,861	27,790	31,153	33,374	35,725	36,665	37,035	37,183	37,244	**-10,114**	**-21.4**
22_HPV_2_25_non	5,521	15,703	22,325	26,918	29,987	31,965	33,998	34,795	35,113	35,245	35,302	**-12,056**	**-25.5**
23_HPV_2_25_64	5,521	16,776	25,494	32,234	37,175	40,635	44,490	46,059	46,654	46,879	46,966	**-392**	**-0.8**
24_HPV_2_25_74	5,521	15,962	23,070	28,130	31,591	33,877	36,298	37,268	37,650	37,804	37,868	**-9,490**	**-20.0**
25_HPV_3_20_non	5,521	17,106	25,841	32,159	36,457	39,244	42,091	43,184	43,611	43,786	43,860	**-3,498**	**-7.4**
26_HPV_3_20_64	5,521	17,912	28,381	36,585	42,588	46,760	51,360	53,215	53,919	54,184	54,289	6,931	14.6
27_HPV_3_20_74	5,521	17,292	26,405	33,111	37,748	40,811	44,015	45,272	45,761	45,956	46,037	**-1,321**	**-2.8**
28_HPV_3_25_non	5,521	17,155	26,000	32,433	36,825	39,680	42,604	43,729	44,169	44,349	44,426	**-2,932**	**-6.2**
29_HPV_3_25_64	5,521	17,961	28,540	36,859	42,956	47,197	51,877	53,768	54,488	54,760	54,868	7,510	15.9
30_HPV_3_25_74	5,521	17,341	26,564	33,385	38,116	41,247	44,529	45,818	46,320	46,521	46,605	**-753**	**-1.6**
31_HPV_5_20_non	5,521	18,636	30,336	39,519	46,082	50,471	55,028	56,760	57,416	57,674	57,782	10,424	22.0
32_HPV_5_20_64	5,521	19,173	32,149	42,830	50,825	56,433	62,624	65,117	66,063	66,420	66,559	19,201	40.5
33_HPV_5_20_74	5,521	18,755	30,716	40,189	47,020	51,638	56,509	58,396	59,113	59,393	59,508	12,150	25.7
34_HPV_5_25_non	5,521	18,667	30,444	39,717	46,364	50,821	55,463	57,234	57,905	58,170	58,281	10,923	23.1
35_HPV_5_25_64	5,521	19,204	32,256	43,028	51,108	56,784	63,062	65,597	66,561	66,926	67,068	19,710	41.6
36_HPV_5_25_74	5,521	18,786	30,824	40,387	47,303	51,988	56,945	58,871	59,604	59,890	60,008	12,650	26.7

[a] Primary screening test_Screening interval_Starting age_Stopping age

[b] Difference compared with the current screening strategy in the National Cancer Screening Program (1_PAP_2_20_non)

© 2025 Luu XQ et al. *JAMA Network Open*.

eTable 6. Cumulative Cervical Cancer Deaths From 2024 to 2100 According to Screening Strategies in Realistic Scenarios

Strategies[a]	2025	2030	2035	2040	2045	2050	2060	2070	2080	2090	2100	Diff[b] (No.)	Diff[b] (%)
1_PAP_2_20_non	1,093	6,426	11,419	15,938	19,884	23,215	28,123	31,132	32,848	33,770	34,242	Ref	Ref
2_PAP_2_20_64	1,093	6,476	11,714	16,656	21,114	24,959	30,704	34,207	36,159	37,180	37,690	3,448	10.1
3_PAP_2_20_74	1,093	6,438	11,485	16,086	20,121	23,537	28,579	31,666	33,418	34,356	34,834	592	1.7
4_PAP_2_25_non	1,093	6,429	11,437	15,982	19,960	23,326	28,300	31,362	33,117	34,066	34,555	313	0.9
5_PAP_2_25_64	1,093	6,479	11,731	16,699	21,190	25,070	30,881	34,439	36,431	37,479	38,007	3,765	11.0
6_PAP_2_25_74	1,093	6,441	11,502	16,129	20,197	23,648	28,755	31,896	33,688	34,653	35,148	906	2.6
7_PAP_3_20_non	1,093	6,496	11,815	16,889	21,510	25,533	31,633	35,454	37,651	38,836	39,441	5,200	15.2
8_PAP_3_20_64	1,093	6,532	12,041	17,462	22,520	26,997	33,866	38,161	40,595	41,879	42,524	8,282	24.2
9_PAP_3_20_74	1,093	6,505	11,863	17,000	21,695	25,790	32,007	35,900	38,133	39,333	39,944	5,702	16.7
10_PAP_3_25_non	1,093	6,498	11,827	16,921	21,569	25,622	31,781	35,652	37,887	39,097	39,718	5,476	16.0
11_PAP_3_25_64	1,093	6,534	12,053	17,494	22,578	27,086	34,014	38,361	40,833	42,143	42,804	8,562	25.0
12_PAP_3_25_74	1,093	6,507	11,875	17,033	21,753	25,878	32,155	36,098	38,369	39,594	40,221	5,979	17.5
13_PAP_5_20_non	1,093	6,563	12,239	17,990	23,504	28,497	36,373	41,460	44,423	46,022	46,836	12,594	36.8
14_PAP_5_20_64	1,093	6,587	12,392	18,396	24,242	29,597	38,120	43,637	46,828	48,530	49,385	15,143	44.2
15_PAP_5_20_74	1,093	6,569	12,270	18,065	23,632	28,680	36,650	41,799	44,796	46,410	47,230	12,988	37.9
16_PAP_5_25_non	1,093	6,565	12,247	18,012	23,544	28,561	36,486	41,617	44,614	46,237	47,065	12,823	37.4
17_PAP_5_25_64	1,093	6,588	12,400	18,418	24,283	29,661	38,233	43,796	47,021	48,747	49,616	15,374	44.9
18_PAP_5_25_74	1,093	6,570	12,278	18,087	23,672	28,744	36,763	41,957	44,987	46,625	47,459	13,217	38.6
19_HPV_2_20_non	1,093	6,358	11,037	15,040	18,379	21,098	24,961	27,246	28,516	29,186	29,524	**-4,718**	**-13.8**
20_HPV_2_20_64	1,093	6,420	11,393	15,883	19,787	23,059	27,790	30,571	32,073	32,839	33,214	**-1,027**	**-3.0**

© 2025 Luu XQ et al. *JAMA Network Open*.

Strategies[a]	2025	2030	2035	2040	2045	2050	2060	2070	2080	2090	2100	Diff[b] (No.)	Diff[b] (%)
21_HPV_2_20_74	1,093	6,373	11,119	15,220	18,662	21,476	25,482	27,847	29,154	29,839	30,183	**-4,059**	**-11.9**
22_HPV_2_25_non	1,093	6,361	11,060	15,096	18,475	21,237	25,174	27,518	28,831	29,530	29,887	**-4,354**	**-12.7**
23_HPV_2_25_64	1,093	6,424	11,416	15,939	19,884	23,197	28,003	30,844	32,391	33,188	33,583	**-659**	**-1.9**
24_HPV_2_25_74	1,093	6,377	11,142	15,277	18,759	21,615	25,695	28,118	29,469	30,184	30,547	**-3,695**	**-10.8**
25_HPV_3_20_non	1,093	6,440	11,452	15,960	19,861	23,124	27,870	30,732	32,342	33,198	33,632	**-610**	**-1.8**
26_HPV_3_20_64	1,093	6,486	11,736	16,660	21,067	24,836	30,403	33,748	35,588	36,540	37,011	2,769	8.1
27_HPV_3_20_74	1,093	6,451	11,514	16,101	20,089	23,434	28,309	31,247	32,892	33,762	34,202	**-40**	**-0.1**
28_HPV_3_25_non	1,093	6,442	11,469	16,004	19,939	23,238	28,053	30,971	32,622	33,506	33,957	**-285**	**-0.8**
29_HPV_3_25_64	1,093	6,489	11,753	16,704	21,145	24,951	30,587	33,989	35,871	36,851	37,340	3,098	9.0
30_HPV_3_25_74	1,093	6,454	11,531	16,144	20,167	23,549	28,493	31,486	33,172	34,071	34,528	286	0.8
31_HPV_5_20_non	1,093	6,523	11,941	17,152	21,910	26,048	32,287	36,153	38,352	39,526	40,122	5,880	17.2
32_HPV_5_20_64	1,093	6,553	12,141	17,673	22,841	27,408	34,379	38,701	41,127	42,398	43,032	8,790	25.7
33_HPV_5_20_74	1,093	6,530	11,982	17,250	22,075	26,279	32,627	36,560	38,793	39,981	40,582	6,340	18.5
34_HPV_5_25_non	1,093	6,524	11,952	17,183	21,967	26,135	32,435	36,352	38,590	39,790	40,402	6,160	18.0
35_HPV_5_25_64	1,093	6,555	12,152	17,703	22,897	27,495	34,528	38,902	41,368	42,665	43,315	9,073	26.5
36_HPV_5_25_74	1,093	6,532	11,993	17,281	22,132	26,366	32,775	36,759	39,031	40,245	40,863	6,621	19.3

[a] Primary screening test_Screening interval_Starting age_Stopping age

[b] Difference compared with the current screening strategy in the National Cancer Screening Program (1_PAP_2_20_non)

© 2025 Luu XQ et al. *JAMA Network Open*.

eTable 7. Projected Total Cervical Cancer Cases and Cases Averted by 2100

Strategies[a]	Scenario		Averted cervical cancer cases[b]	
	Realistic	Ideal	No.	%
1_PAP_2_20_non	47,358	39,522	7,836	17%
2_PAP_2_20_64	57,354	52,247	5,107	9%
3_PAP_2_20_74	49,410	43,531	5,879	12%
4_PAP_2_25_non	47,906	40,373	7,533	16%
5_PAP_2_25_64	57,915	53,119	4,796	8%
6_PAP_2_25_74	49,960	44,387	5,573	11%
7_PAP_3_20_non	58,708	49,514	9,194	16%
8_PAP_3_20_64	67,380	61,088	6,292	9%
9_PAP_3_20_74	60,407	53,253	7,154	12%
10_PAP_3_25_non	59,202	50,356	8,846	15%
11_PAP_3_25_64	67,884	61,949	5,935	9%
12_PAP_3_25_74	60,903	54,100	6,803	11%
13_PAP_5_20_non	74,202	64,064	10,139	14%
14_PAP_5_20_64	81,160	73,921	7,239	9%
15_PAP_5_20_74	75,501	67,348	8,154	11%
16_PAP_5_25_non	74,620	64,872	9,748	13%
17_PAP_5_25_64	81,585	74,746	6,838	8%
18_PAP_5_25_74	75,920	68,160	7,760	10%
19_HPV_2_20_non	34,681	28,883	5,798	17%
20_HPV_2_20_64	46,329	42,838	3,491	8%
21_HPV_2_20_74	37,244	33,147	4,097	11%
22_HPV_2_25_non	35,302	29,750	5,552	16%
23_HPV_2_25_64	46,966	43,729	3,237	7%
24_HPV_2_25_74	37,868	34,020	3,848	10%
25_HPV_3_20_non	43,860	36,537	7,322	17%
26_HPV_3_20_64	54,289	49,606	4,683	9%
27_HPV_3_20_74	46,037	40,620	5,417	12%
28_HPV_3_25_non	44,426	37,391	7,035	16%
29_HPV_3_25_64	54,868	50,481	4,386	8%
30_HPV_3_25_74	46,605	41,480	5,125	11%
31_HPV_5_20_non	57,782	48,680	9,102	16%
32_HPV_5_20_64	66,559	60,350	6,209	9%
33_HPV_5_20_74	59,508	52,443	7,065	12%
34_HPV_5_25_non	58,281	49,523	8,758	15%
35_HPV_5_25_64	67,068	61,213	5,855	9%
36_HPV_5_25_74	60,008	53,291	6,717	11%

[a] Primary screening test_Screening interval_Starting age_Stopping age
[b]Adverted cervical cancer cases by increasing HPV vaccination and cervical cancer screening rate to 90% and 70% from 2030

eTable 8. Projected Total Cervical Cancer Deaths and Deaths Averted by 2100

Strategies[a]	Scenario		Averted cervical cancer deaths[b]	
	Realistic	Ideal	No.	%
1_PAP_2_20_non	34,242	31,149	3,093	9%
2_PAP_2_20_64	37,690	35,270	2,420	6%
3_PAP_2_20_74	34,834	32,204	2,629	8%
4_PAP_2_25_non	34,555	31,635	2,920	8%
5_PAP_2_25_64	38,007	35,763	2,244	6%
6_PAP_2_25_74	35,148	32,692	2,455	7%
7_PAP_3_20_non	39,441	35,572	3,869	10%
8_PAP_3_20_64	42,524	39,435	3,089	7%
9_PAP_3_20_74	39,944	36,594	3,350	8%
10_PAP_3_25_non	39,718	36,047	3,671	9%
11_PAP_3_25_64	42,804	39,916	2,888	7%
12_PAP_3_25_74	40,221	37,070	3,151	8%
13_PAP_5_20_non	46,836	42,336	4,500	10%
14_PAP_5_20_64	49,385	45,734	3,651	7%
15_PAP_5_20_74	47,230	43,270	3,960	8%
16_PAP_5_25_non	47,065	42,785	4,280	9%
17_PAP_5_25_64	49,616	46,188	3,429	7%
18_PAP_5_25_74	47,459	43,720	3,739	8%
19_HPV_2_20_non	29,524	27,350	2,174	7%
20_HPV_2_20_64	33,214	31,569	1,646	5%
21_HPV_2_20_74	30,183	28,382	1,801	6%
22_HPV_2_25_non	29,887	27,855	2,033	7%
23_HPV_2_25_64	33,583	32,080	1,502	4%
24_HPV_2_25_74	30,547	28,888	1,659	5%
25_HPV_3_20_non	33,632	30,692	2,940	9%
26_HPV_3_20_64	37,011	34,722	2,289	6%
27_HPV_3_20_74	34,202	31,705	2,497	7%
28_HPV_3_25_non	33,957	31,186	2,772	8%
29_HPV_3_25_64	37,340	35,221	2,119	6%
30_HPV_3_25_74	34,528	32,199	2,329	7%
31_HPV_5_20_non	40,122	36,245	3,877	10%
32_HPV_5_20_64	43,032	39,920	3,112	7%
33_HPV_5_20_74	40,582	37,204	3,378	8%
34_HPV_5_25_non	40,402	36,725	3,677	9%
35_HPV_5_25_64	43,315	40,405	2,910	7%
36_HPV_5_25_74	40,863	37,685	3,178	8%

[a] Primary screening test_Screening interval_Starting age_Stopping age
[b]Adverted cervical cancer deaths by increasing HPV vaccination and cervical cancer screening rate to 90% and 70% from 2030

eFigure 1. Simplified Structure of the Model

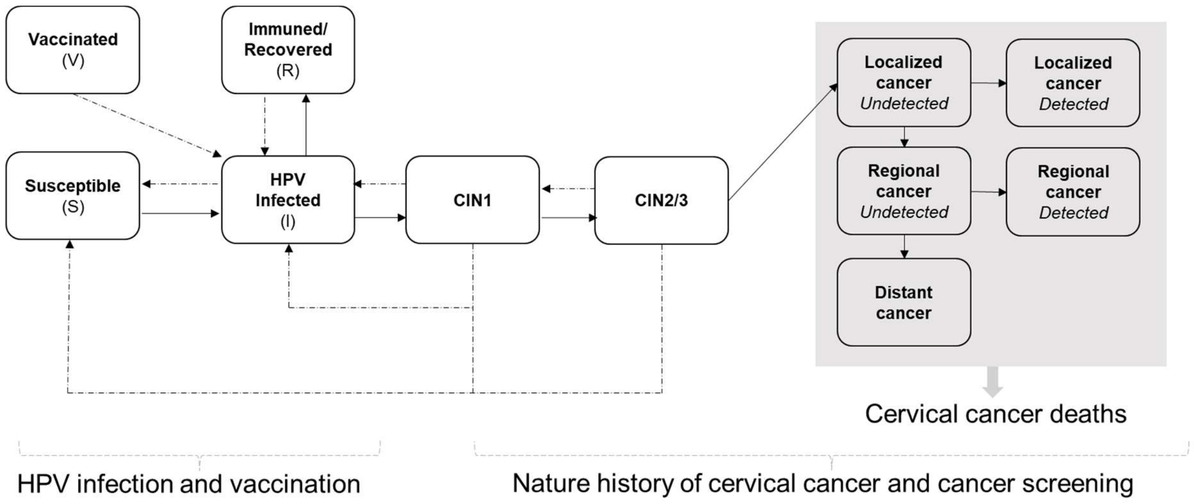

© 2025 Luu XQ et al. *JAMA Network Open*.

eFigure 2. Population Size Over the Projected Period Compared With KOSIS Data and Projection

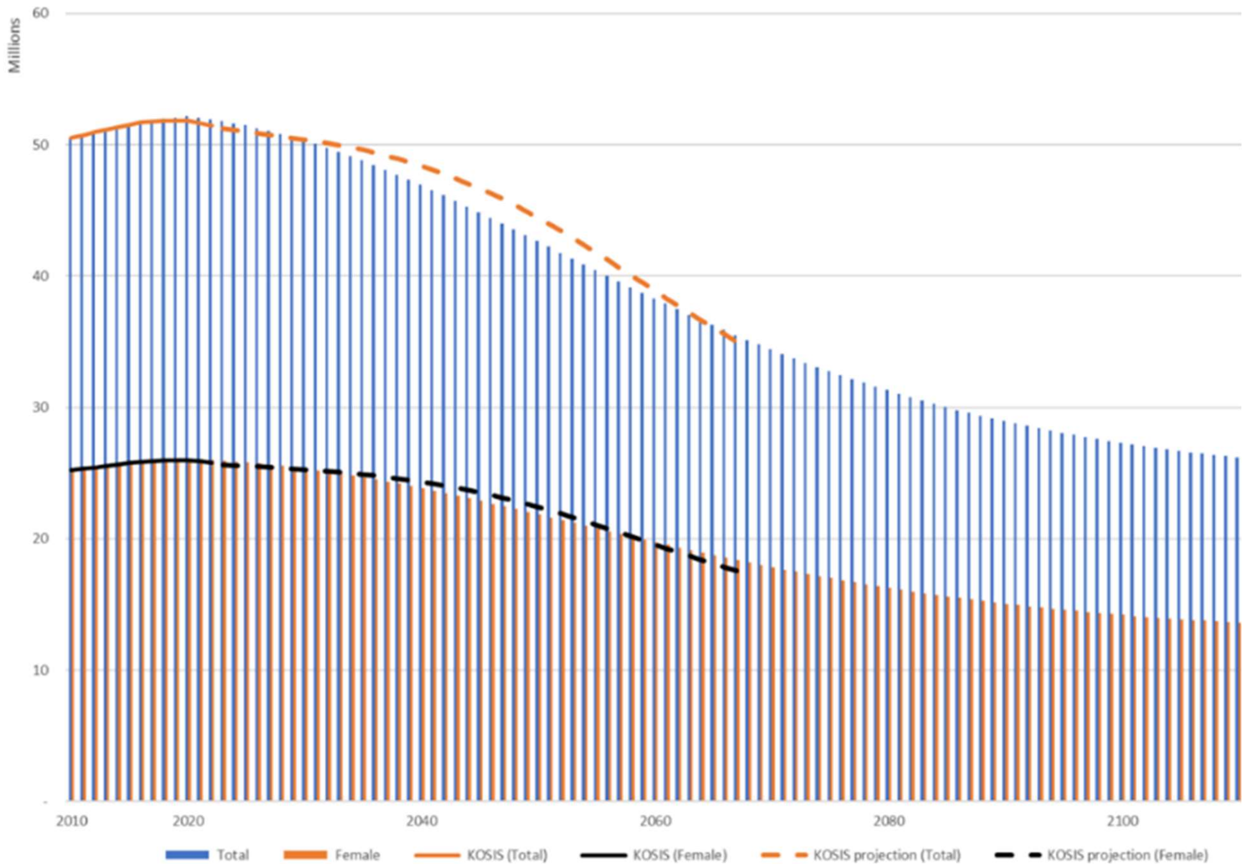

© 2025 Luu XQ et al. *JAMA Network Open*.

eFigure 3. Trend In Age-Standardized Cervical Cancer Incidence According to Screening Strategies Toward Cervical Cancer Elimination Under Realistic Scenarios

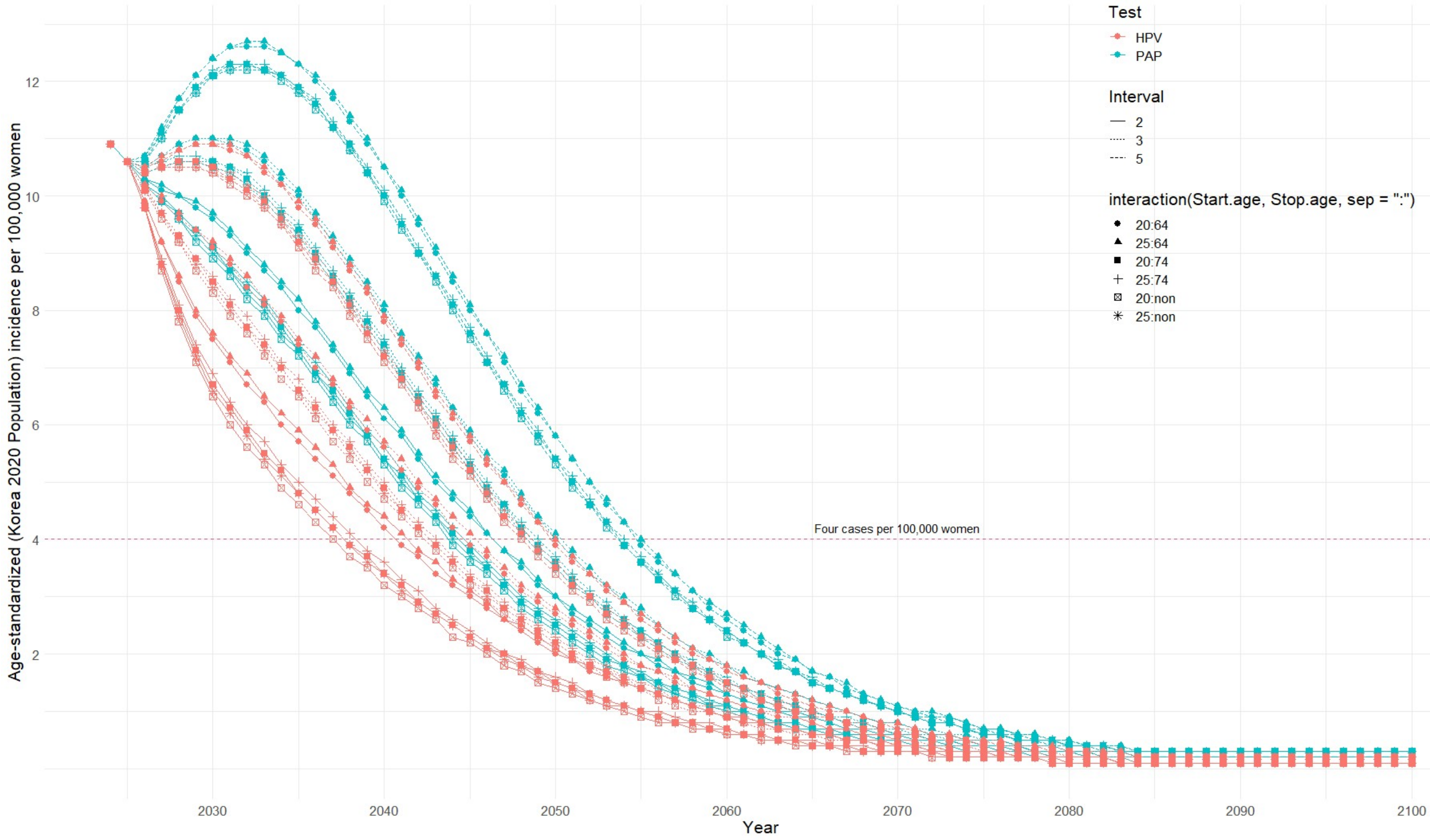

© 2025 Luu XQ et al. *JAMA Network Open*.

eFigure 4. Trend In Age-Standardized Cervical Cancer Incidence According to Screening Strategies Using Pap Smear Under Realistic Scenarios

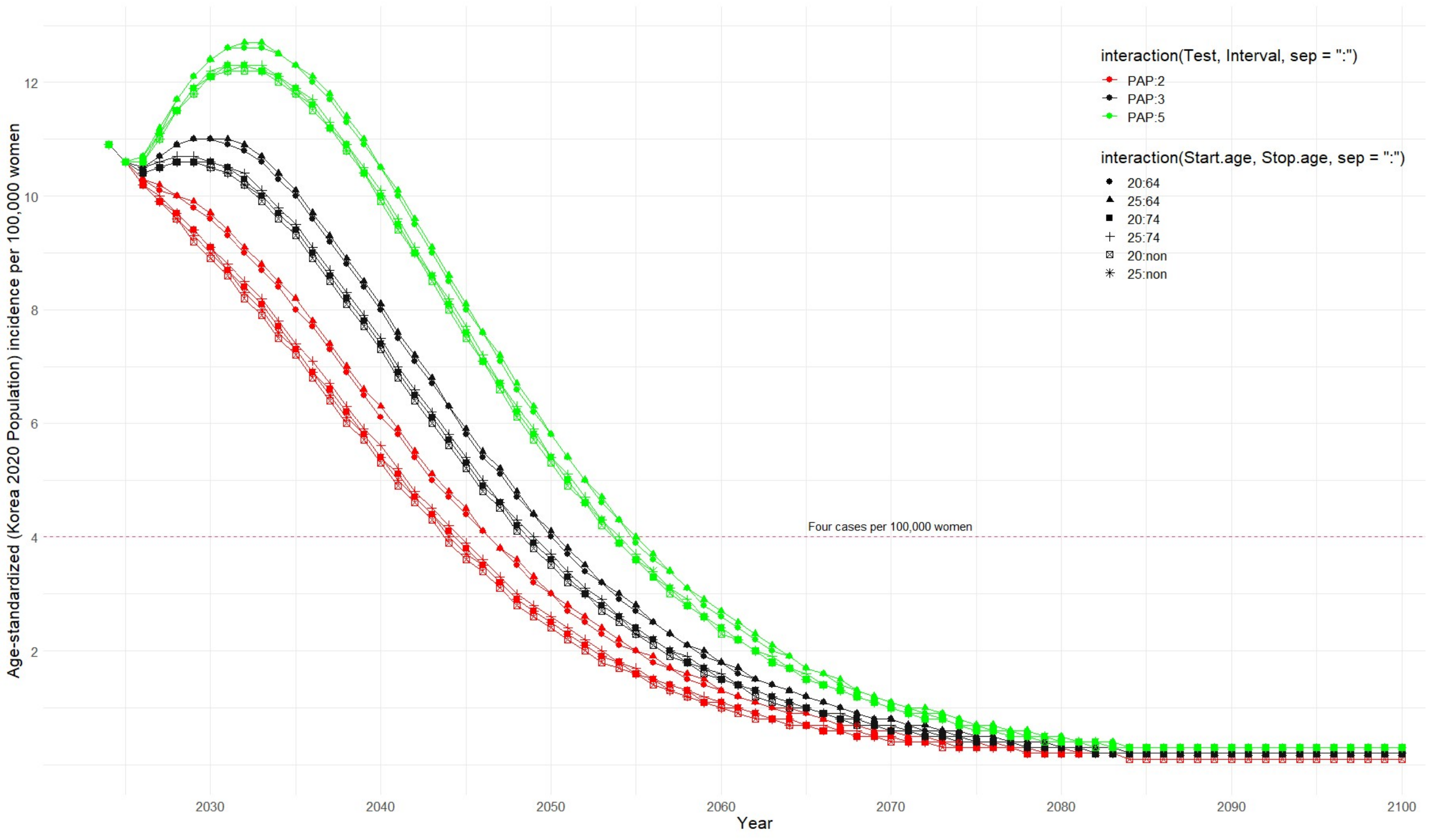

© 2025 Luu XQ et al. *JAMA Network Open*.

eFigure 5. Trend In Age-Standardized Cervical Cancer Incidence According to Screening Strategies Using HPV Testing Under Realistic Scenarios

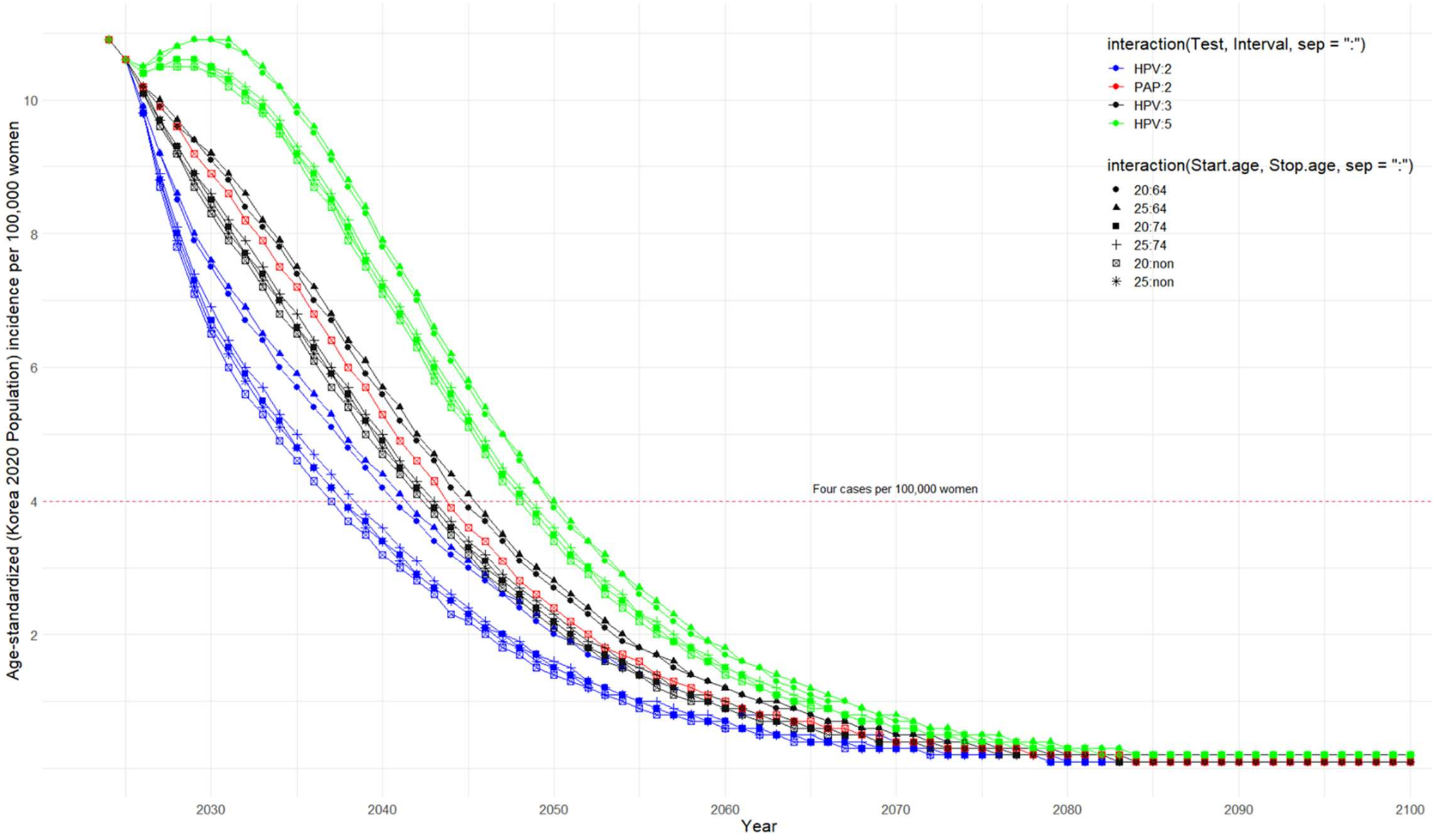

© 2025 Luu XQ et al. *JAMA Network Open*.

eReferences.

1. Choi JY, Kim M, Kwon B-S, Jeong SJ, Suh DH, Kim K, et al. Human papillomavirus vaccine uptake in South Korea. CEOG. 2022;49(1).
2. NECA. Cost-effectiveness analysis for expanding national immunization program against HPV. 2023.
3. Ahmad OB, Boschi-Pinto C, Lopez AD, Murray CJ, Lozano R, Inoue M. Age standardization of rates: a new WHO standard. Geneva: World Health Organization. 2001;9(10):1-14.
4. Resident Population in Five-Year Age Groups [Internet]. Korean Statistical Information Service. [cited 2023/05/18]. Available from: https://kosis.kr/statHtml/statHtml.do?orgId=101&tblId=DT_1B04005N&conn_path=I2&language=en.
5. Total Fertility Rates and Age-Specific Fertility Rates [Internet]. Korean Statistical Information Service. [cited 2023/05/18]. Available from: https://kosis.kr/statHtml/statHtml.do?orgId=101&tblId=DT_1B81A21&conn_path=I2&language=en.
6. Deaths by Age and Sex [Internet]. Korean Statistical Information Service. [cited 2023/05/18]. Available from: https://kosis.kr/statHtml/statHtml.do?orgId=101&tblId=DT_1B80A11&conn_path=I2&language=en.
7. Campos NG, Burger EA, Sy S, Sharma M, Schiffman M, Rodriguez AC, et al. An updated natural history model of cervical cancer: derivation of model parameters. Am J Epidemiol. 2014;180(5):545-55.
8. Durham DP, Ndeffo-Mbah ML, Skrip LA, Jones FK, Bauch CT, Galvani AP. National- and state-level impact and cost-effectiveness of nonavalent HPV vaccination in the United States. Proceedings of the National Academy of Sciences. 2016;113(18):5107-12.
9. Luu XQ, Lee K, Jun JK, Suh M, Jung KW, Lim MC, et al. Effect of Pap smears on the long-term survival of cervical cancer patients: a nationwide population-based cohort study in Korea. Epidemiol Health. 2022;44:e2022072.
10. Mayrand M-H, Duarte-Franco E, Rodrigues I, Walter SD, Hanley J, Ferenczy A, et al. Human Papillomavirus DNA versus Papanicolaou Screening Tests for Cervical Cancer. New England Journal of Medicine. 2007;357(16):1579-88.
11. Bui CN, Choi E, Suh M, Jun JK, Jung KW, Lim MC, et al. Trend analysis of process quality indicators for the Korean National Cervical Cancer Screening Program from 2005 to 2013. J Gynecol Oncol. 2021;32(1).
12. Kim MA, Oh JK, Chay DB, Park DC, Kim SM, Kang ES, et al. Prevalence and seroprevalence of high-risk human papillomavirus infection. Obstet Gynecol. 2010;116(4):932-40.
13. Clifford GM, Shin HR, Oh JK, Waterboer T, Ju YH, Vaccarella S, et al. Serologic response to oncogenic human papillomavirus types in male and female university students in Busan, South Korea. Cancer Epidemiol Biomarkers Prev. 2007;16(9):1874-9.
14. KCDC. HPV vaccination status. Osong: Korea Disease Control and Prevention Agency; 2023.

© 2025 Luu XQ et al. *JAMA Network Open*.
