# Supplementary material for: Cervical Cancer Screening, HPV Vaccination, and Cervical Cancer Elimination
Source: JAMA Netw Open. 2025 Aug 12;8(8):e2526683. doi: 10.1001/jamanetworkopen.2025.26683 (PMC12344532; doi:10.1001/jamanetworkopen.2025.26683)
Supplement: Supplement 2. — Data Sharing Statement [file jamanetwopen-e2526683-s002.pdf]

Data Sharing Statement

Luu. Cervical Cancer Screening, HPV Vaccination, and Cervical Cancer Elimination. *JAMA Netw Open*. Published August 12, 2025. doi:10.1001/jamanetworkopen.2025.26683

Data
Data available: Yes
Data types: Data dictionary
How to access data: e-mail: lxq@huph.edu.vn
When available: With publication

Supporting Documents
Document types: Statistical/analytic code
How to access documents: e-mail: lxq@huph.edu.vn
When available: With publication

Additional Information
Who can access the data: Researchers whose proposed use of the data has been approved
Types of analyses: Research purpose
Mechanisms of data availability: With investigator support
